# Do offspring characteristics reflect parental migration variation?

**DOI:** 10.1111/jfb.70247

**Published:** 2025-10-04

**Authors:** Madeleine Berry, Jan G. Davidsen, Marie Nevoux, Kim Aarestrup, Carlos M. Alexandre, Sara S. Silva, Alexander Thorén, Anders Engstöm, Matilda Ahvenainen, Johan Höjesjö

**Affiliations:** ^1^ Department of Biological and Environmental Sciences University of Gothenburg Gothenburg Sweden; ^2^ Department of Natural History NTNU University Museum Trondheim Norway; ^3^ DECOD L'Institut Agro, IFREMER, INRAE Rennes France; ^4^ Pôle MIAME (Management of Diadromous Fish in their Environment), OFB, INRAE, UPPA L'Institut Agro Rennes France; ^5^ Section for Freshwater Fisheries and Ecology Technical University of Denmark Silkeborg Denmark; ^6^ MARE – Centro de Ciências do Mar e do Ambiente/ARNET – Rede de Investigação Aquática, Instituto de Investigação e Formação Avançada Universidade de Évora Évora Portugal

**Keywords:** behaviour, boldness, Europe, migration, morphology, salmonid

## Abstract

Sea trout, *Salmo trutta*, display a wide range of migratory behaviours, and one aspect of variation comes from freshwater migration distance. The overall aim of this study was to determine if offspring of long‐ and short‐distance migrants exhibited phenotypic differences relating to parental migration distance. For that purpose, we conducted several behavioural tests (dyadic contest, boldness scoring and open field test) and morphological analysis (relative pectoral‐fin length) in multiple freshwater systems across the distribution range of the target species in Europe. It was expected that offspring of long‐distance migrants would be more active, bold and dominant than those of short‐distance migrants and would have longer pectoral fins relative to body length. Additionally, we investigated if boldness varied in relation to latitude. We showed that offspring of long‐distance migrants were more dominant in two cases and more active in one case than those of short‐distance migrants; however, there was no difference in swimming distance or velocity. Boldness and relative pectoral‐fin length were significantly related to site of origin; however, the direction of this relationship differed between systems. Generally, we detected a decrease in boldness with declining latitude. In summary, we have detected variation among juveniles related to location within a stream; however, the drivers and processes behind these are likely more complex than purely parental migratory strategy. Our results can inform suitable management and conservation efforts directed to anadromous *Salmo trutta*. For example, habitat restoration and removal of migration barriers can increase the possible range of migration distances helping maintain the phenotypic diversity of offspring.

## 1 INTRODUCTION

Migration patterns vary widely among species, both temporally and spatially. These different patterns can profoundly influence population dynamics, as well as various aspects of an organism's biology and, ultimately, it's reproductive success (Fayet et al., 2017; Reid et al., 2020). For migratory fishes, such as salmonids, migration behaviours can dictate patterns of gene flow and local adaptations through occupation of different geographic locations (Rougemont et al., 2023). In sea trout (the anadromous form of brown trout), *Salmo trutta* L., the length of both marine and freshwater migrations is highly variable (Östergren et al., 2011; Strøm et al., 2021). In the case of upstream freshwater migrations, individuals with short migration distances may benefit from reduced energetic costs and decreased exposure to predators, whereas those undertaking long‐distance migrations may reach better‐quality spawning grounds or areas with more favourable environmental conditions and fewer competitors (Bohlin et al., 2001; Brönmark et al., 2014; Nevoux et al., 2019).

With increased migration distance comes increased challenge, and this variability in migration distance is influenced by factors such as the availability of suitable spawning habitat (Zimmer & Power, 2006), energetic cost (Kristensen et al., 2011) and the presence of physical barriers such as dams, weirs or waterfalls (Gosset et al., 2006). The cost of migration could potentially separate the type of individuals undergoing long migrations from those completing short migrations. These differences may extend to offspring due to genetic, maternal or environmental influences, leading to distinct juvenile phenotypes (Höjesjö et al., 2011; Stelkens et al., 2012). A recent study demonstrated such spatial sorting of physiological characteristics in juvenile *S. trutta* (Berry et al., 2024), but potentially behavioural and morphological characteristics could also play a role.

Behavioural traits, such as boldness or tendency to partake in risky behaviour, can impact migration distance such that bolder individuals are more likely to explore novel environments and, in general, bolder fish are more prone to disperse further (Cote et al., 2010; Fraser et al., 2001). In European eels, *Anguilla anguilla*, individuals from upstream sites show more risky explorative behaviours than their downstream counterparts (Podgorniak et al., 2016). Similarly, in Atlantic salmon, *Salmo salar*, behavioural type influences how individuals cope when navigating hydropower stations (Haraldstad et al., 2019, 2021). To complete a long migration requires adults to be bold – to explore new areas – as well as active – to get there. These differences may also be passed down to offspring resulting in shyer, less active, individuals being produced downstream compared to upstream. Aggression has previously been shown to differ with migratory strategy; for example, *S. trutta* from populations with longer migrations (sea‐run) are more aggressive than those with shorter migrations (lake‐run) and resident populations (Lahti et al., 2001, 2002). To compete for and hold higher‐quality spawning sites, adults need to be dominant, and less‐competitive individuals may end up being forced out of high‐quality sites, further shaping the phenotypic characteristics of subsequent offspring. This spatial sorting of behavioural traits results from the assumption that generally substrate structure is more suitable for spawning upstream with greater occurrences of gravel beds. In addition, increases in organic matter downstream can cause embeddedness reducing suitability of sites downstream.

Morphological characteristics may also contribute to the ability of individuals to migrate further. Larger pectoral fins give fish an advantage when swimming, and in salmonids, pectoral fins are used for manoeuvring, holding position and braking (Drucker & Lauder, 2003), making them an important feature for negotiating obstacles. In coho salmon, *Oncorhynchus kisutch*, a comparison between coastal and inland populations in the same river system revealed differences in overall body shape and fin sizes in juveniles and adults (Taylor & McPhail, 1985). Furthermore, the same study showed that these differences were inherited by offspring when reared under equal conditions in a laboratory setting.

Spatial sorting can function as a form of selection, dictating how distributions of traits vary across space (Bowler & Benton, 2005; Phillips & Perkins, 2019). *S. trutta* display strong natal homing (Ferguson et al., 2019; Jonsson & Jonsson, 2014), with individuals often returning to the same reach or tributary, although straying is known to occur (Källo et al., 2022). Despite extensive research on the migratory behaviour of salmonids, to our knowledge, little attention has been given to the potential effect of variation in parental migration distance on the distribution of their offspring's phenotypic traits. Moreover, little or no research has addressed such issues at wide‐range scale, covering the full latitudinal gradient of the target species. Latitudinal gradients are known to structure behavioural and physiological traits across taxa. Behavioural traits, including boldness, have been shown to vary with latitude in birds (Díaz et al., 2013), fish (Culumber, 2022) and amphibians (Cortazar‐Chinarro et al., 2025). In *S. trutta* traits such as metabolic rate (Lahti et al., 2002), energy state (Finstad et al., 2010) and growth (Jensen et al., 2000) vary with latitude. These traits could link to differences in boldness as they can influence risk‐taking, competitive ability and overall behavioural strategies (Finstad et al., 2007; Metcalfe et al., 1995). This suggests that environmental factors associated with latitude (e.g., photoperiod, temperature and length of growing season) may contribute to larger geographic patterns of juvenile boldness variation.

In this paper, we present the findings of a comprehensive study examining differences in juvenile anadromous *S. trutta* originating from long‐ and short‐distance migrant parents in six different *S. trutta* systems across the native range in Europe. Site of capture serves as a proxy for site of origin, given the limited dispersal of trout in the juvenile phase (Höjesjö et al., 2015; Palm et al., 2023; Vøllestad et al., 2012), thus upstream and downstream sites were used to categorize offspring of long‐ and short‐distance migrants. By combining behavioural studies and morphometric analyses, we sought to determine how parental migratory distance shapes the behavioural tendencies and physical attributes of their offspring. It was predicted that offspring of long‐distance migrants would be more bold, more dominant, more active and have longer pectoral fins relative to body length than offspring of short‐distance migrants due to genetic, maternal and/or environmental effects associated with the site of origin. Given the large geographic range of sites used within this study, we also aim to investigate if upstream/downstream spatial patterns of boldness vary within the native range along a latitudinal gradient. These findings could have important implications for anadromous *S. trutta* conservation in the face of ongoing environmental changes across Europe by advancing our understanding of how spawning migration distance variation influences offspring characteristics.

## 2 METHODS

### 2.1 Sampling

**FIGURE 1:**
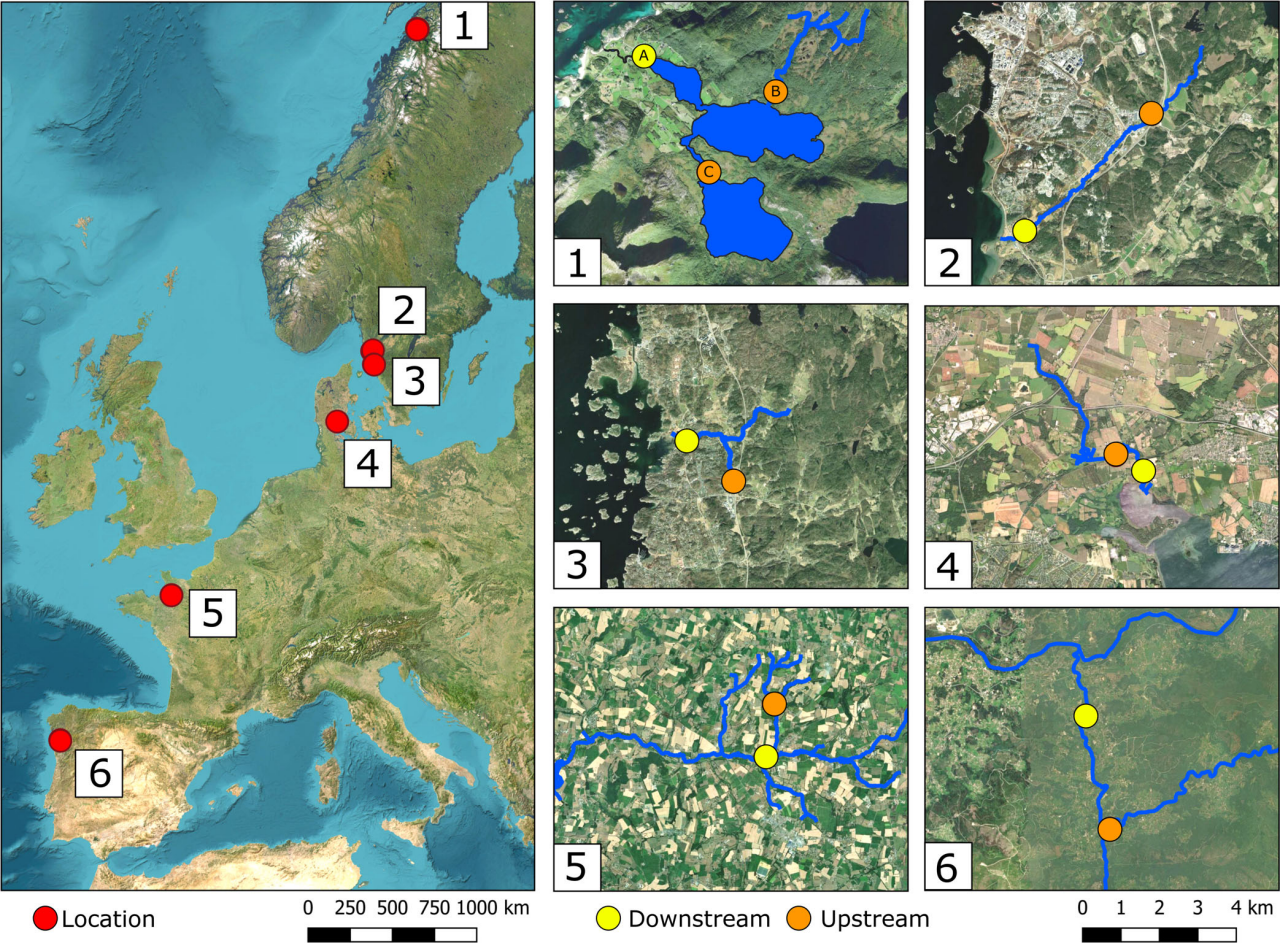
Maps of the six *Salmo trutta* study locations. The left panel shows their positions within Europe (red circles). The right panels show each site with the stream systems outlined in blue (the stream systems are not plotted in their entirety); downstream sites are depicted with yellow circles and upstream sites with orange circles (1: Fjærvatnet with sites labelled A–C, 2: Norumsån, 3: Haga å, 4: Gudsø, 5: La Roche, 6: Mouro). Produced using QGIS with ESRI Satellite (ArcGIS/World Imagery) used as a basemap.

Six locations across the anadromous *S. trutta* range were used in this study, covering the full latitudinal gradient of the species' natural distribution in Europe (Figure 1). At each location, an upstream and a downstream site were selected, separated by at least 1 km. A variety of behavioural tests were conducted, including boldness scoring, open field tests and dyadic contests. Logistical constraints determined which experiments could be conducted at each site; a summary of the tests and sample sizes is provided in Table 1 (more detailed site‐specific information can be found in Appendix A).

**TABLE 1:** Table displaying which studies were conducted at which locations (Fjærvatnet, Norumsån, Haga å, Gudsø, La Roche, Mouro) with associated sample sizes of *Salmo trutta*.

	Boldness scoring		Open field test		Dyadic contest		Pectoral‐fin length	
	Down	Up	Down	Up	Down	Up	Down	Up
1: Fjærvatnet (Norway)	48	49, 15	–	–	–	–	47	92, 15
2: Norumsån (Sweden)	–	–	51	51	50	50	–	–
3: Haga å (Sweden)	30	30	36	50	32	32	32	170
4: Gudsø (Denmark)	51	44	–	–	–	–	110	90
5: La Roche (France)	49	65	–	–	–	–	–	–
6: Mouro (Portugal)	20	20	–	–	–	–	–	–

*Note:* Each location is separated into two sites; downstream (Down) and upstream (Up).

Electrofishing was conducted at each site targeting juvenile trout (parr stage). Captured fish were kept in boxes supplied with a constant flow of stream water prior to sampling. For the open field tests and dyadic contests, fish were transported to laboratory facilities at the University of Gothenburg. After behavioural testing, fish were returned to their respective streams for release. Boldness scoring was conducted in the field. After scoring, fish were anaesthetised (benzocaine; 0.5 mL L^−1^), weighed, fork length measured and, in some cases, pectoral‐fin length also measured. Relative pectoral‐fin length was calculated as: pectoral‐fin length (cm)/fork length (cm) × 100. After a recovery period, fish were released back at their site of capture.

### 2.2 Dyadic contest

A dyadic contest, in which pairs of fish engage in competitive interactions to assess dominance (Höjesjö et al., 2002), was used to compare individuals from upstream and downstream sites. For this purpose, fish from Norumsån and Haga å were transported to laboratory facilities at the University of Gothenburg. Here, they were transferred into aerated 10°C holding aquaria with three sides covered, a flow‐through water system, gravel substrate and plastic refugia objects. Fish were fed daily with chironomid larvae. After a minimum of 48 h of acclimatization, they were anaesthetised (2‐phenoxyethanol 0.5 mL L^−1^ or benzocaine; 0.5 mL L^−1^), fork length and wet body mass measured, and a 12‐mm PIT tag was inserted into the abdomen via a small incision behind the left pectoral fin for individual identification. After the fish were tagged, they returned to the holding tanks and allowed to recover for 6 days before the first behavioural observations.

The contests were performed with length‐matched pairs of one individual from each site, with a maximal size difference of 5% (Huntingford et al., 1990). This size matching resulted in 50 unique pairs from Norumsån and 28 unique pairs from Haga å. Fish were identified with PIT tags (Haga å), and their adipose fins were clipped in different directions in a randomized manner (Norumsån). Aquaria 28 × 20 cm were set up in arrays of eight to ten with removable dividing walls placed in the middle of the aquaria, creating two separate compartments and supplied with water from the same source as the holding tanks. For Haga å trials, four cameras were mounted above the contest aquaria. To encourage interactions within the pairs, feeding was ceased 2 days before initiating the trials. Twelve (Norumsån) or 22 (Haga å) h prior to trials, pairs were placed into the separate compartments of the contest aquaria to acclimate.

After acclimation, contests were initiated by removing the dividing wall. The first aquaria were observed for 5 min before initiating the contest in the next aquaria. This procedure was repeated for the remaining aquaria. Each interaction was recorded or observed until a set social rank could be observed with a time limit of maximum 30 min (Haga å) or 20 min (Norumsån). The social rank was established by a number of social interactions: attack, chasing, flee, display, swimming, brightest colouration, holding low and holding high (Keenleyside & Yamamoto, 1962). The individual that displayed the most agnostic behaviours during the trial was established as the dominant fish.

### 2.3 Open field test

Open field tests are used to assess activity (% of time spent active), swimming speed (cm/s) and swimming distance (cm) in fish when tracked in a barren arena (Burns, 2008). Fish from Norumsån and Haga å were assessed in this manner. Boxes (65 × 48 cm) were set up in arrays of six to eight with two cameras mounted above and filled with 5–10 cm water, and custom light ramps were used to eliminate shading. Fish were randomly selected and placed into tanks individually and recorded for 35 (Norumsån) or 25 (Haga å) min. Water was replaced between each trial.

All films were subsequently edited using either VideoPad version 6.29 NCH to remove the first and last 3 min (Norumsån) or AVI Splitter version 2.21 BRIZ Software to remove the first and last 5 min (Haga å) when fish were likely disturbed. This was then analysed using tracking software (LoliTrack 4.0 Loligo Systems ApS, Viborg, Denmark).

### 2.4 Boldness scoring

To minimize transportation stress and to facilitate the testing of many individuals within a short time frame, a simple test of boldness was used. Sudden exposure to light in a novel environment was used to elicit a startle response, serving as a proxy for boldness (Berry et al., 2025). Fish were selected at random and transferred to a small opaque round box (diameter = 30 cm) filled with 10 cm of water from the stream. The boxes were arranged in arrays of four and positioned under a tripod‐mounted camera and covered with opaque plastic lids that blocked all light. After a 2‐min acclimation period, the covers were removed, and the fish were recorded for 5 min. Water was replaced between each trial. Video footage was later analysed manually, and time until the first movement (s) following sudden exposure to light was measured (for Gudsø, time was measured with a human observer rather than video recording). Individuals that did not move during the trial were categorized as shy, whereas those that moved were classified as bold. Among the bold individuals, latency (in seconds) until first movement was used to further differentiate boldness, with shorter latencies indicating higher boldness.

### 2.5 Statistical analyses

All statistical analysis was performed using RStudio version 2023.03.0 (http://www.R-project.org/). Normality was checked using Shapiro–Wilk tests. Outcomes of the dyadic trials were analysed using binomial tests; one dyad from the Haga å sample was excluded as a fish escaped the tank. In both dyadic contest studies, one trial was excluded after a ‘winner’ could not be established.

For open field tests activity (%), swimming speed and distance were analysed using Kruskall–Wallis tests due to non‐normal distributions. For each sample (Norumsån and Haga å), the variables were tested individually against the site of origin.

Data on relative pectoral‐fin length were collected from Fjærvatnet, Haga å and Gudsø. Each sample was analysed using Kruskall–Wallis tests due to non‐normal distributions, comparing relative pectoral‐fin length and site of origin (upstream/downstream). For Fjærvatnet, because there were more than two locations, a Dunn post hoc test was performed using the R package FSA, with *p*‐values adjusted using the Benjamini‐Hochberg method to determine the relationship between groups.

For the boldness scoring, the proportion of bold and shy individuals was assessed using a generalized linear model with a binomial distribution and logit link function, including site within stream, stream system and fork length (to rule out any differences relating to size); an interaction between site and stream system was also included. Estimated marginal means and pair‐wise contrasts of boldness between upstream and downstream fish within each stream system were assessed using the R package emmeans (Lenth, 2025), and odds ratios were used to evaluate the direction and strength of differences (odds ratios >1 indicating higher boldness upstream and < 1 indicating higher boldness downstream).

To investigate any general relationship with latitude, an additional generalized model with binomial distribution and logit function was made with bold/shy outcome, latitude and fork length as a covariate to control for body size. For bold fish, the boldness score (s) was analysed using a Kruskall–Wallis test; for the Fjærvatnet sample, additional analysis was conducted using a Dunn post hoc test, with *p*‐values adjusted with the Benjamini–Hochberg method.

## 3 RESULTS

### 3.1 Dyadic contest

**FIGURE 2:**
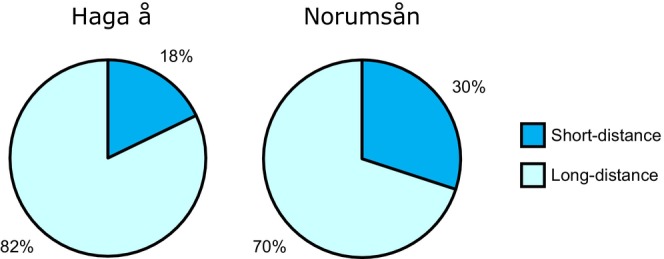
Pie charts displaying proportion of winners in dyadic contests from *Salmo trutta* pairs of offspring of long‐ and short‐distance migrants. The left chart shows results of fish sampled from Haga å, and the right chart shows results of fish sampled from Norumsån. Dark blue segments represent short‐distance winners, and light blue segments represent long‐distance winners; percentages are also displayed.

In the dyadic contest of fish from Norumsån, offspring of long‐distance migrants were more likely to win in trials than those of short‐distance migrants [95% confidence interval (CI) (0.554, 0.821), *p* = 0.007] and won in 35 out of 50 (70%) trials (Figure 2). In the dyadic contest of fish from Haga å, offspring of long‐distance migrants were more likely to win in trials than offspring of short‐distance migrants [95% CI (0.631, 0.939), *p* < 0.001] and won in 23 out of 28 (82%) trials (Figures 2).

### 3.2 Open field test

In the open field test conducted with fish from Haga å, activity (% of time spent active) was significantly higher in fish from the upstream site (offspring of long‐distance migrants). There was no significant difference in swimming speed (cm/s) or distance travelled (cm) between offspring of long‐distance and short‐distance migrants (Table 2). In the open field test conducted with fish from Norumsån, there was no significant difference in activity (%), swimming speed (cm/s) or distance travelled (cm) between offspring of long‐distance and short‐distance migrants, although there is a trend in the Norumsån sample of upstream fish swimming slower and covering less distance (Table 2).

**TABLE 2:** Means, standard deviations and *p*‐values (significant *p*‐values are highlighted in bold) for open field tests from *Salmo trutta* originating upstream and downstream in Haga å and Norumsån.

	Haga å			Norumsån		
	Downstream	Upstream	*p*‐Value	Downstream	Upstream	*p*‐Value
Activity (%)	60.1 ± 9.3	65.5 ± 8.8	**0.03**	38.3 ± 18.6	35.0 ± 18.7	0.36
Swimming speed (cm/s)	11.8 ± 2.9	12.0 ± 2.8	0.72	4.5 ± 2.4	4.0 ± 2.1	0.07
Distance (m)	43.2 ± 12.5	46.8 ± 11.8	0.20	88.3 ± 51.1	73.6 ± 48.4	0.08

### 3.3 Boldness scoring

**FIGURE 3:**
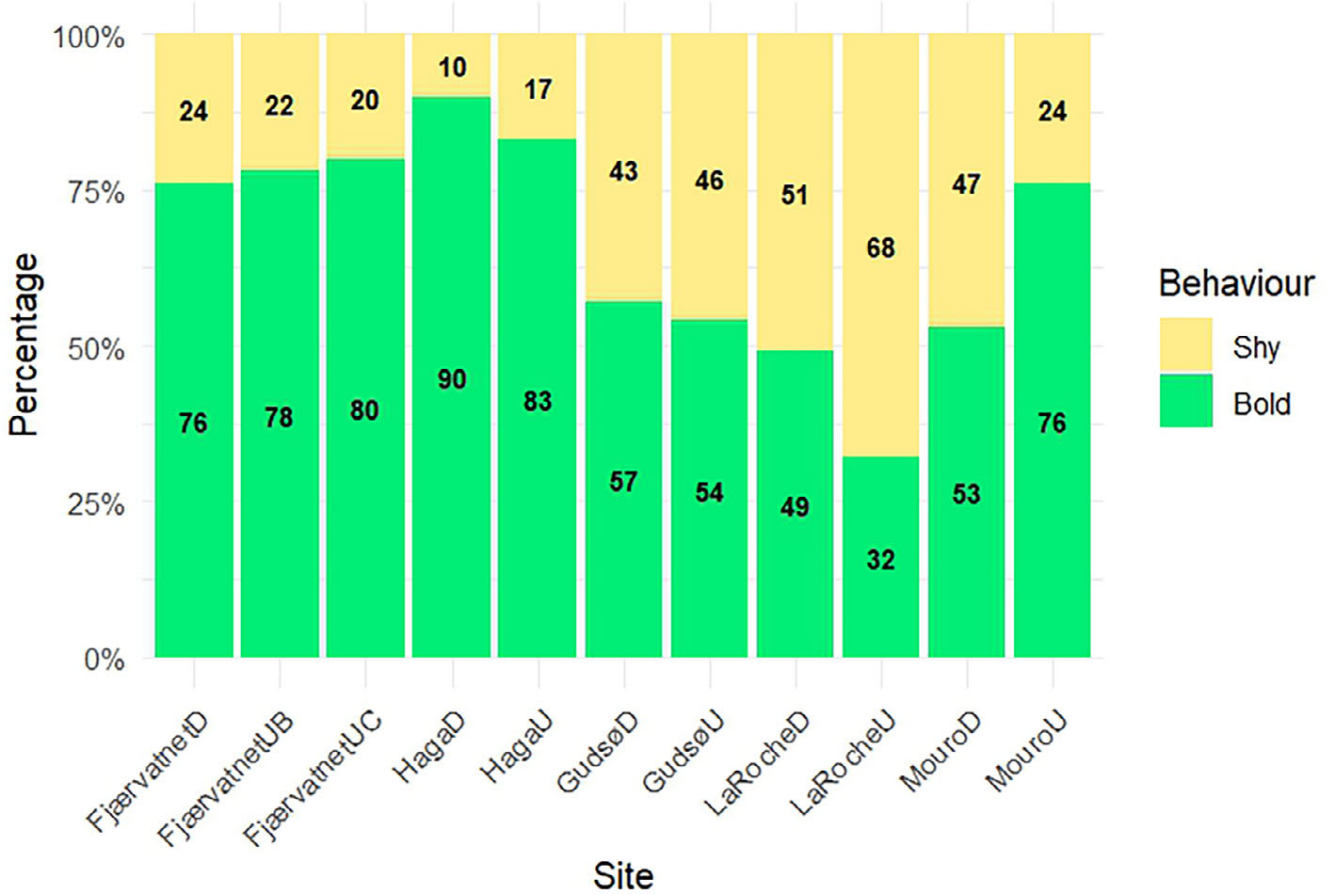
Stacked barplot showing the proportion of bold (green) and shy (yellow) *Salmo trutta* individuals at each site. Actual percentages are displayed on the graph. Sites are indicated in the *x‐*axis labels, with ‘D’ and ‘U’ indicating downstream and upstream sites, respectively (note at Fjærvatnet, there are two upstream sites labelled UB and UC).

When considering the bold/shy outcome, the interaction between site within stream and stream system was significant [*β* = 1.2, standard error (SE) = 0.6, *z* = 2.0, *p* = 0.05], indicating upstream/downstream effects differed between locations. The estimated marginal means suggested a trend in La Roche for a higher proportion of bold individuals downstream compared to upstream. Although this effect was non‐significant (odds ratio = 0.5, *p* = 0.09), there was also no significant effect at the other locations: Fjærvatnet (odds ratio = 1.2, *p* = 0.7), Haga å (odds ratio = 0.5, *p* = 0.4), Gudsø (odds ratio = 1.7, *p* = 0.2), Mouro (odds ratio = 3.0, *p* = 0.1). Additionally, no effect of fork length was found (*β* = −0.02, SE = 0.04, *z* = −0.5, *p* = 0.6). Further investigation showed a significant relationship between the proportion of shy and bold individuals and latitude (Figure 3), with the prevalence of boldness decreasing with decreasing latitude (estimate = −0.07, SE = 0.01, *z* = −5.0, *p* < 0.001), and there was no significant effect of fork length (estimate = −0.03, SE = 0.03, *z* = −1.1, *p* = 0.3).

**FIGURE 4:**
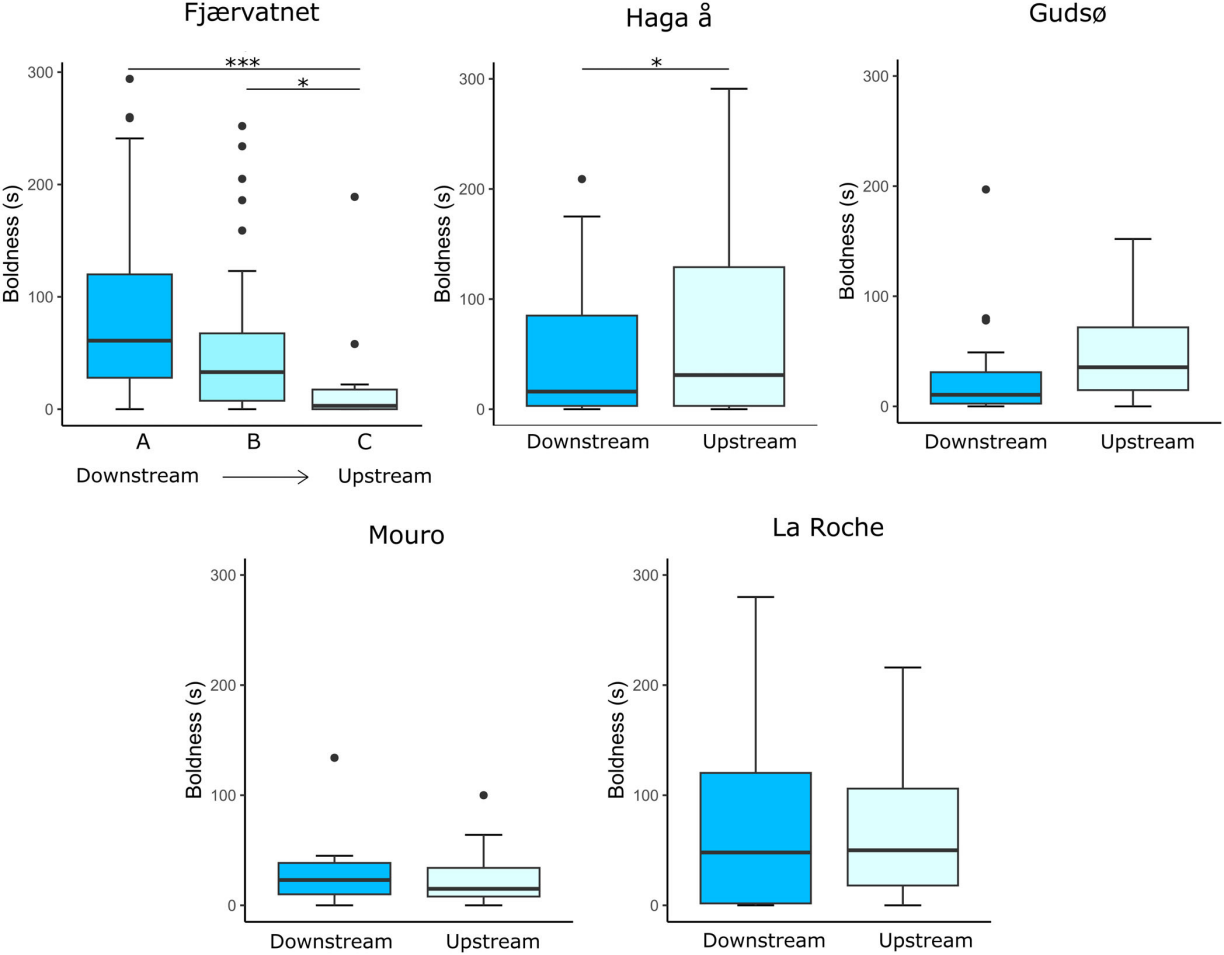
Boxplots showing the relationships between site and boldness (seconds until first movement) in *Salmo trutta*. The top left panel shows the sample from Fjærvatnet, with distance to sea increasing from A to C. The middle top panel shows the sample from Haga å, the top right panel from Gudsø, bottom left from Mouro and bottom right from La Roche. The horizontal line in each box indicates the median, and the box boundaries indicate the interquartile range. Outliers are displayed with black dots, and significant group differences are indicated with horizontal bars and asterisks.

Within fish categorized as bold, there was some variation in degree of boldness (measured as latency until first movement). In Fjærvatnet, there was a significant difference in boldness (s) between the three sites in individuals that did move during the trial period (Kruskal–Wallis *χ* ^2^ = 14.2, *p* < 0.001), with boldness decreasing from A to C (Figure 4). Individuals from site C exhibited significantly shorter latencies and were therefore bolder than individuals from sites A (*z* = 3.7, *p* < 0.001) and B (*z* = 2.4, *p* = 0.02); individuals from sites A and B were not significantly different but followed a similar trend (*z* = 2.0, *p* = 0.06). In Haga å, offspring of short‐distance migrants were significantly bolder (shorter latencies) than those of long‐distance migrants (Kruskal–Wallis *χ* ^2^ = 5.0, *p* = 0.03). In Gudsø, there was no difference in time until movement in bold fish between sites (Kruskal–Wallis *χ* ^2^ = 0.4, *p* = 0.5). In La Roche, there was no difference in time until movement in bold fish between sites (Kruskal–Wallis *χ* ^2^ = 0.2, *p* = 0.7). In Mouro, there was no difference in time until movement in bold fish between sites (Kruskal–Wallis *χ* ^2^ = 0.08, *p* = 0.8).

### 3.4 Morphology

**FIGURE 5:**
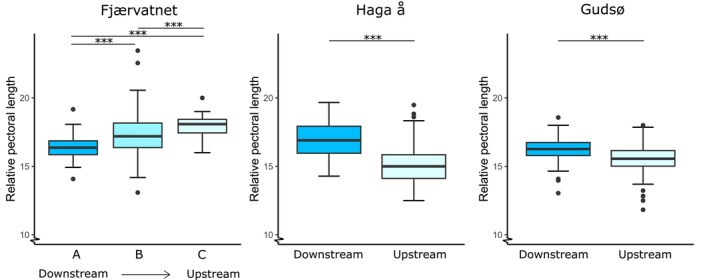
Boxplots showing the relationship between site and relative pectoral‐fin length of *Salmo trutta*. The left panel shows the sample from Fjærvatnet. The middle panel shows the sample from Haga å and the right panel from Gudsø. The horizontal line in each box indicates the median, and the box boundaries indicate the interquartile range. Outliers are displayed with black dots, and significant group differences are displayed with horizontal bars and asterisks.

In Fjærvatnet, relative pectoral‐fin length had a significant relationship with site (Kruskal–Wallis *χ* ^2^ = 28.9, *p* < 0.001) and increased from site A to C (Figure 5); individuals from site A had smaller fins relative to body size compared to sites B (*z* = −4.2, *p* < 0.001) and C (*z* = −4.7, *p* < 0.001); individuals from site B had smaller fins than site C (*z* = −2.3, *p* < 0.001) (Figure 5). In Haga å, fish originating downstream had longer relative pectoral fins (Kruskal–Wallis *χ* ^2^ = 34.4, *p* < 0.001) (Figure 5). In Gudsø, fish originating from downstream had relatively longer pectoral fins than those originating upstream (Kruskal–Wallis *χ* ^2^ = 28.4, *p* < 0.001).

## 4 DISCUSSION

In this paper, we have demonstrated that morphological characteristics and behavioural properties might differ between offspring of long‐ and short‐distance migrants, although, interestingly, not always in the same direction. In the case of dominance, we see strong evidence of an upstream–downstream relationship with more dominant individuals being found upstream in two cases. However, for other traits, such as boldness and relative pectoral‐fin length, we see a lack of consistent directional patterns emphasizing the complexity of the relationship between site of origin and offspring traits. We cannot rule out other underlying factors that might act together or oppose the upstream–downstream relationship. Behavioural and morphological characteristics can be determined by both genetic and environmental factors and their interaction (Dermond et al., 2019). Even within the same stream system, juveniles may have different genetic backgrounds (Stelkens et al., 2012) and may experience different environmental conditions, for example, in flow regime, water chemistry, shear stress, resource availability, among others. However, using trout as a model organism where the offspring generally stays close to the spawning site allows us to integrate both of these factors due to the genetics and maternal influences of the adults and the variability in habitat due to spawning site selection (Foldvik et al., 2010; Serbezov et al., 2010). The degree of habitat variability between upstream and downstream sites differs between the six localities in this study, as well as the total freshwater migration distance from sea. These differences have not been quantified presently, and it is unknown to what extent the phenotypic variations explored here are driven by genetics or environmental factors (e.g., microhabitats).

One of the most significant and novel findings of this study is that offspring of long‐distance migrants were consistently more dominant than those of short‐distance migrants. This result, demonstrated across two stream systems (Haga å and Norumsån), highlights a crucial difference between juveniles originating in different stream sections. Being more dominant could give individuals a competitive advantage during the juvenile stage, which will likely impact future survival and fitness. Notably, previous studies have contradictory findings, in which juvenile *S. trutta* from a downstream section were more aggressive than upstream (Kristensen & Closs, 2008); however, in their study the residency/anadromy differed between upstream and downstream populations due to an impassable migration barrier between the two sites resulting in a resident population upstream and a mixed resident/migrant population downstream (Kristensen et al., 2011). Open field tests showed activity was significantly higher in offspring of long‐distance migrants than short‐distance migrants in the sample from Haga å, in keeping with our expectations, but not the sample from Norumsån. There was also no significant relationship between site of origin and swimming distance and swimming speed. Given the plastic nature of *S. trutta*, these parameters may differ on a larger geographic scale and may be more similar in juveniles produced within the same stream systems (Meier et al., 2011). Further studies could investigate whether these traits differ between sites within streams separated by larger distances or with more environmental heterogeneity.

In the case of boldness, our results revealed a more complex pattern. Although we did not observe a clear relationship between upstream and downstream localities at the extreme ends of the scale (bold vs. shy fish), we did find significant differences in degree of boldness within bold individuals. In Fjærvatnet, within bold individuals, offspring of long‐distance migrants were bolder than those of short‐distance migrants. However, in the Haga å and Gudsø systems, the reverse relationship was found: offspring of short‐distance migrants were bolder than those of long‐distance migrants. This intriguing finding still supports variation among juvenile characteristics at small spatial scales within streams but hints that perhaps factors other than parentage may be drivers, for example, population densities or habitat characteristics. These factors are certainly worth investigating further. When considering Haga å, where most studies were conducted, the patterns of dominance/activity and boldness revealed a complex pattern and were not consistent with our expectations, suggesting a weak or context‐specific link between boldness and competitive interaction.

The presence of a significant trend associated with boldness and latitude suggests that factors such as climate or latitude‐related ecological pressures may influence boldness in juvenile *S. trutta*. One possible explanation is that populations at higher latitudes compensate for the shorter growing season by increasing risk‐taking and adopting bolder foraging strategies, a pattern consistent with the concept of countergradient variation. Countergradient variation suggests that growth rate should increase as the growing season decreases and has been demonstrated in several fish species (Yamahira et al., 2007; Yamahira & Conover, 2002). Under the assumption that being more bold enhances food intake and therefore growth (Jolles et al., 2016; Stamps, 2007), it could be an important mechanism by which *S. trutta* at higher latitudes achieve sufficient growth during a short growing season. Although this trend in boldness requires further investigation, it raises broader questions about the large‐scale environmental gradients that shape behavioural traits.

Regarding pectoral‐fin morphology, in Fjærvatnet we observed a general trend of relative pectoral‐fin length increasing by an average 10% between the sites closest to and furthest from the sea; however, it is important to bear in mind that the sample size at site C was much smaller than desirable (*n* = 15). However, the large relative difference (>10%) reported suggests that the size of pectoral fin could be of high biological relevance. The reverse relationship was found in both Haga å and Gudsø. In Haga å, for example, offspring of short‐distance migrants had an average 13% longer pectoral fins relative to body length, again suggesting a relationship between site of origin and offspring characteristics, but also indicates that other factors may be the driving force. Clearly, there does not need to be a linear relationship between the relative length of the pectoral fin and the migratory distance per se, but rather an adaptation to local conditions (Páez et al., 2010). In agreement, pectoral‐fin length has previously been shown to differ relating to local hydraulic conditions, where individuals living in areas prone to high discharge had larger pectoral fins (Drinan et al., 2012). Future studies can also investigate other aspects of morphology, including the size of other fins, body shape and general body condition, in relation to local conditions at various distances from the sea. Our results also suggest a possible relationship between relative pectoral‐fin length and boldness, though this was not formally tested and would require further investigation.

Anadromous *S. trutta* utilize many different freshwater systems, from small transient streams to large river systems hundreds of kilometres long, as well as lake systems. Here, we studied juveniles from several stream systems of varying length and a system with lakes present (Fjærvatnet). Local adaptation may differ between different types of systems, particularly for a species with a flexible life history such as *S. trutta*. Resident, lake‐migrating and sea migrating trout exhibit vastly different morphological and behavioural traits (Lahti et al., 2001; Taylor & McPhail, 1985). Therefore, it is crucial to acknowledge that variations observed in one system may not necessarily reflect those in other systems. However, the main insight is that juvenile trout may vary locally based on their site of origin. Now that differences have been established, it would be of great interest to conduct further studies using common garden experiments, removing the influence of environmental variability, to further investigate the drivers of this variation. Repeated sampling over time and space, as well as increasing the distance between the upstream and downstream sites or working on gradients of sites, would enhance the detection of differences and thus improve the robustness of the approach.

In this study, juvenile parentage was assumed by site of capture, although it has not been confirmed that juveniles used in the various studies are progeny of parents of different migratory strategies. However, previous studies have reported that even when juvenile salmonids are transplanted, they generally are very sedentary, remaining within 40 m of the release point for several months and showing no or a limited tendency of homing behaviour. This supports our assumption that juvenile *S. trutta* have limited dispersal before migration, making the site of capture equivalent to the site of origin (Foldvik et al., 2010; Höjesjö et al., 2015). Additionally, we cannot rule out the occurrence of residency in our study populations (Ferguson et al., 2019; Sahashi & Morita, 2013); however, to the best of our knowledge, all sites selected were composed predominantly of anadromous populations.

In short, offspring characteristics do not simply reflect parental dispersal distances; however, there are indications of spatial variation in phenotypic characteristics within a system. For some traits, such as dominance, there seems to be a more consistent upstream–downstream pattern. However other traits, such as boldness and relative pectoral‐fin length, vary in both directions. These findings hint at the possible importance of local adaptations and/or maternal influences on variation in juvenile phenotypic traits. Freshwater systems are subject to increasing anthropogenic pressures that could influence the distribution patterns of anadromous *S. trutta* (Ayllón et al., 2016). The presence of dams and other human infrastructure instream, as well as the escalating risk of drought due to climate change, impacts the structure and connectivity of freshwater systems. Juvenile phenotypic variation could be essential for population resilience in the face of changing environments, as it provides a broader range of phenotypes that may be advantageous under different ecological pressures.

## AUTHOR CONTRIBUTIONS

Madeleine Berry, Jan G. Davidsen, Marie Nevoux, Kim Aarestrup, Carlos M. Alexandre and Johan Höjesjö developed the research plan. Madeleine Berry, Sara S. Silva, Alexander Thorén, Anders Engstöm and Matilda Ahvenainen participated in data collection. Madeleine Berry analysed the data. Madeleine Berry led the writing of the manuscript and Jan G. Davidsen, Marie Nevoux, Kim Aarestrup, Carlos M. Alexandre, Sara S. Silva, Alexander Thorén, Anders Engstöm, Matilda Ahvenainen and Johan Höjesjö contributed to the development of the manuscript.

## FUNDING INFORMATION

The work was funded by a grant from FORMAS (grant no: 2020‐01349).

## APPENDIX A

### A.1 SAMPLING ADDITIONAL DETAILS

**Table:**

Site	Period	Approximate distance to sea from downstream site (to nearest km)	Approximate distance between downstream and upstream sites	Total number of fish (downstream, upstream)	Fork lengths, mean ± standard deviation (mm)
*Norumsån* *(58.04318° N, 11.84589° E)*	September 2018	1 km	6 km	50, 50	117 ± 15
*Haga å (57.57542° N, 11.94316° E)*	September 2021 and 2022	1 km	2.5 km	2021: 52, 412,022: 32, 174	2021: 96 ± 3 2022: 85 ± 9
*Gudsø (55.53057° N, 9.57667° E)*	September 2021	1 km	1.5 km	110, 90	150 ± 15
*La Roche (48.63873° N, 1.17502° W)*	September 2022	20 km	1.3 km	49, 65	120 ± 43
*Fjærvatnet (67.50271° N, 14.72136° E)*	September 2022	1 km	3 km	54 (A), 94 (B), 15 (C)	117 ± 23
*Mouro (42.05854° N, 8.38914° W)*	September 2023	50 km	5 km	20, 20	149 ± 18

## References

[jfb70247-bib-0001] Ayllón, D. , Railsback, S. F. , Vincenzi, S. , Groeneveld, J. , Almodóvar, A. , & Grimm, V. (2016). InSTREAM‐gen: Modelling eco‐evolutionary dynamics of trout populations under anthropogenic environmental change. Ecological Modelling, 326, 36–53. 10.1016/j.ecolmodel.2015.07.026

[jfb70247-bib-0002] Berry, M. , Austad, B. , & Höjesjö, J. (2025). Streamlining boldness measurement in fish: A practical approach to field studies. Behavioural Processes, 226, 105162. 10.1016/j.beproc.2025.105162 39914615

[jfb70247-bib-0003] Berry, M. , Zena, L. A. , Roques, J. A. C. , Sandblom, E. , Thorstad, E. B. , & Höjesjö, J. (2024). Local variation in stress response of juvenile anadromous brown trout, *Salmo trutta* . Ecology and Evolution, 14(6), e11526. 10.1002/ece3.11526 38932968 PMC11199126

[jfb70247-bib-0004] Bohlin, T. , Pettersson, J. , & Degerman, E. (2001). Population density of migratory and resident brown trout (*Salmo trutta*) in relation to altitude: Evidence for a migration cost: Altitude and population density of brown trout. Journal of Animal Ecology, 70(1), 112–121. 10.1111/j.1365-2656.2001.00466.x

[jfb70247-bib-0005] Bowler, D. E. , & Benton, T. G. (2005). Causes and consequences of animal dispersal strategies: Relating individual behaviour to spatial dynamics. Biological Reviews, 80(2), 205–225. 10.1017/S1464793104006645 15921049

[jfb70247-bib-0006] Brönmark, C. , Hulthén, K. , Nilsson, P. A. , Skov, C. , Hansson, L.‐A. , Brodersen, J. , & Chapman, B. B. (2014). There and back again: Migration in freshwater fishes. Canadian Journal of Zoology, 92(6), 467–479. 10.1139/cjz-2012-0277

[jfb70247-bib-0007] Burns, J. G. (2008). The validity of three tests of temperament in guppies (*Poecilia reticulata*). Journal of Comparative Psychology, 122(4), 344–356. 10.1037/0735-7036.122.4.344 19014258

[jfb70247-bib-0008] Cortazar‐Chinarro, M. , Corral‐Lopez, A. , Lüdtke, D. U. , Tegnér, F. , Luquet, E. , & Laurila, A. (2025). Metamorphosis reverses the behavioral phenotype in Rana arvalis along a latitudinal gradient. Ecology and Evolution, 15(8), e71945. 10.1002/ece3.71945 40809828 PMC12344277

[jfb70247-bib-0009] Cote, J. , Clobert, J. , Brodin, T. , Fogarty, S. , & Sih, A. (2010). Personality‐dependent dispersal: Characterization, ontogeny and consequences for spatially structured populations. Philosophical Transactions of the Royal Society, B: Biological Sciences, 365(1560), 4065–4076. 10.1098/rstb.2010.0176 PMC2992741 21078658

[jfb70247-bib-0010] Culumber, Z. W. (2022). Variation in behavioral traits across a broad latitudinal gradient in a livebearing fish. Evolutionary Ecology, 36(1), 75–91. 10.1007/s10682-021-10146-5

[jfb70247-bib-0011] Dermond, P. , Sperlich, N. , & Brodersen, J. (2019). Heritable morphological differentiation in salmonids from two distinct stream types. Journal of Fish Biology, 95(5), 1215–1222. 10.1111/jfb.14121 31418819

[jfb70247-bib-0012] Díaz, M. , Møller, A. P. , Flensted‐Jensen, E. , Grim, T. , Ibáñez‐Álamo, J. D. , Jokimäki, J. , Markó, G. , & Tryjanowski, P. (2013). The geography of fear: A latitudinal gradient in anti‐predator escape distances of birds across Europe. PLoS One, 8(5), e64634. 10.1371/journal.pone.0064634 23724070 PMC3665823

[jfb70247-bib-0013] Drinan, T. J. , McGinnity, P. , Coughlan, J. P. , Cross, T. F. , & Harrison, S. S. C. (2012). Morphological variability of Atlantic salmon *Salmo salar* and brown trout *Salmo trutta* in different river environments. Ecology of Freshwater Fish, 21(3), 420–432. 10.1111/j.1600-0633.2012.00561.x

[jfb70247-bib-0014] Drucker, E. G. , & Lauder, G. V. (2003). Function of pectoral fins in rainbow trout: Behavioral repertoire and hydrodynamic forces. Journal of Experimental Biology, 206(5), 813–826. 10.1242/jeb.00139 12547936

[jfb70247-bib-0015] Fayet, A. L. , Freeman, R. , Anker‐Nilssen, T. , Diamond, A. , Erikstad, K. E. , Fifield, D. , Fitzsimmons, M. G. , Hansen, E. S. , Harris, M. P. , Jessopp, M. , Kouwenberg, A.‐L. , Kress, S. , Mowat, S. , Perrins, C. M. , Petersen, A. , Petersen, I. K. , Reiertsen, T. K. , Robertson, G. J. , Shannon, P. , … Guilford, T. (2017). Ocean‐wide drivers of migration strategies and their influence on population breeding performance in a declining seabird. Current Biology, 27(24), 3871–3878. 10.1016/j.cub.2017.11.009 29199078

[jfb70247-bib-0016] Ferguson, A. , Reed, T. E. , Cross, T. F. , McGinnity, P. , & Prodöhl, P. A. (2019). Anadromy, potamodromy and residency in brown trout *Salmo trutta*: The role of genes and the environment. Journal of Fish Biology, 95(3), 692–718. 10.1111/jfb.14005 31197849 PMC6771713

[jfb70247-bib-0017] Finstad, A. G. , Berg, O. K. , Forseth, T. , Ugedal, O. , & Næsje, T. F. (2010). Adaptive winter survival strategies: Defended energy levels in juvenile Atlantic salmon along a latitudinal gradient. Proceedings of the Royal Society B: Biological Sciences, 277(1684), 1113–1120. 10.1098/rspb.2009.1874 PMC2842767 20007174

[jfb70247-bib-0018] Finstad, A. G. , Forseth, T. , Ugedal, O. , & Næsje, T. F. (2007). Metabolic rate, behaviour and winter performance in juvenile Atlantic salmon. Functional Ecology, 21(5), 905–912. 10.1111/j.1365-2435.2007.01291.x

[jfb70247-bib-0019] Foldvik, A. , Finstad, A. G. , & Einum, S. (2010). Relating juvenile spatial distribution to breeding patterns in anadromous salmonid populations. Journal of Animal Ecology, 79(2), 501–509. 10.1111/j.1365-2656.2009.01652.x 20050942

[jfb70247-bib-0020] Fraser, D. F. , Gilliam, J. F. , & Daley, M. J. (2001). Explaining leptokurtic movement distributions: Intrapopulation variation in boldness and exploration. The American Naturalist, 158(2), 124–135. 10.1086/321307 18707341

[jfb70247-bib-0021] Gosset, C. , Rives, J. , & Labonne, J. (2006). Effect of habitat fragmentation on spawning migration of brown trout (*Salmo trutta* L.). Ecology of Freshwater Fish, 15(3), 247–254. 10.1111/j.1600-0633.2006.00144.x

[jfb70247-bib-0022] Haraldstad, T. , Haugen, T. O. , Kroglund, F. , Olsen, E. M. , & Höglund, E. (2019). Migratory passage structures at hydropower plants as potential physiological and behavioural selective agents. Royal Society Open Science, 6(11), 190989. 10.1098/rsos.190989 31827840 PMC6894575

[jfb70247-bib-0023] Haraldstad, T. , Haugen, T. O. , Olsen, E. M. , Forseth, T. , & Höglund, E. (2021). Hydropower‐induced selection of behavioural traits in Atlantic salmon (Salmo salar). Scientific Reports, 11(1), 16444. 10.1038/s41598-021-95952-1 34385548 PMC8360942

[jfb70247-bib-0024] Höjesjö, J. , Adriaenssens, B. , Bohlin, T. , Jönsson, C. , Hellström, I. , & Johnsson, J. I. (2011). Behavioural syndromes in juvenile brown trout (*Salmo trutta*); life history, family variation and performance in the wild. Behavioral Ecology and Sociobiology, 65(9), 1801–1810. 10.1007/s00265-011-1188-0

[jfb70247-bib-0025] Höjesjö, J. , Johnsson, J. , & Bohlin, T. (2002). Can laboratory studies on dominance predict fitness of young brown trout in the wild? Behavioral Ecology and Sociobiology, 52(2), 102–108. 10.1007/s00265-002-0493-z

[jfb70247-bib-0026] Höjesjö, J. , Johnsson, J. I. , & Bohlin, T. (2015). Behavior and growth of juvenile brown trout (*Salmo trutta*) following upstream and downstream displacement. Journal of Freshwater Ecology, 30(3), 455–461. 10.1080/02705060.2014.971448

[jfb70247-bib-0027] Huntingford, F. A. , Metcalfe, N. B. , Thorpe, J. E. , Graham, W. D. , & Adams, C. E. (1990). Social dominance and body size in Atlantic salmon parr, *Salmo solar* L. Journal of Fish Biology, 36(6), 877–881. 10.1111/j.1095-8649.1990.tb05635.x

[jfb70247-bib-0028] Jensen, A. J. , Forseth, T. , & Johnsen, B. O. (2000). Latitudinal variation in growth of young brown trout *Salmo trutta* . Journal of Animal Ecology, 69(6), 1010–1020. 10.1111/j.1365-2656.2000.00457.x

[jfb70247-bib-0029] Jolles, J. W. , Manica, A. , & Boogert, N. J. (2016). Food intake rates of inactive fish are positively linked to boldness in three‐spined sticklebacks *Gasterosteus aculeatus* . Journal of Fish Biology, 88(4), 1661–1668. 10.1111/jfb.12934 26940195 PMC4982035

[jfb70247-bib-0030] Jonsson, B. , & Jonsson, N. (2014). Naturally and hatchery produced European trout Salmo trutta: Do their marine survival and dispersal differ? Journal of Coastal Conservation, 18(2), 79–87. 10.1007/s11852-012-0224-1

[jfb70247-bib-0031] Källo, K. , Baktoft, H. , Kristensen, M. L. , Birnie‐Gauvin, K. , & Aarestrup, K. (2022). High prevalence of straying in a wild brown trout (*Salmo trutta*) population in a fjord system. ICES Journal of Marine Science, 79(5), 1539–1547. 10.1093/icesjms/fsac079

[jfb70247-bib-0032] Keenleyside, M. H. A. , & Yamamoto, F. T. (1962). Territorial behaviour of juvenile Atlantic Salmon (*Salmo salar* L.). Behaviour, 19(1–2), 139–168. 10.1163/156853961X00231

[jfb70247-bib-0033] Kristensen, E. A. , & Closs, G. P. (2008). Variation in growth and aggression of juvenile brown trout (Salmo trutta) from upstream and downstream reaches of the same river. Ecology of Freshwater Fish, 17(1), 130–135. 10.1111/j.1600-0633.2007.00266.x

[jfb70247-bib-0034] Kristensen, E. A. , Closs, G. P. , Olley, R. , Kim, J. , Reid, M. , & Stirling, C. (2011). Determining the spatial distribution of spawning by anadromous and resident brown trout Salmo trutta L using strontium content of eggs collected from redds: Strontium content of brown trout eggs. Ecology of Freshwater Fish, 20(3), 377–383. 10.1111/j.1600-0633.2010.00451.x

[jfb70247-bib-0035] Lahti, K. , Huuskonen, H. , Laurila, A. , & Piironen, J. (2002). Metabolic rate and aggressiveness between Brown trout populations. Functional Ecology, 16(2), 167–174. 10.1046/j.1365-2435.2002.00618.x

[jfb70247-bib-0036] Lahti, K. , Laurila, A. , Enberg, K. , & Piironen, J. (2001). Variation in aggressive behaviour and growth rate between populations and migratory forms in the brown trout, *Salmo trutta* . Animal Behaviour, 62(5), 935–944. 10.1006/anbe.2001.1821

[jfb70247-bib-0037] Lenth, R. (2025). emmeans: Estimated Marginal Means, aka Least‐Squares Means (Version R package version 1.11.1‐00001) [Computer software]. https://rvlenth.github.io/emmeans/

[jfb70247-bib-0038] Meier, K. , Hansen, M. M. , Bekkevold, D. , Skaala, Ø. , & Mensberg, K.‐L. D. (2011). An assessment of the spatial scale of local adaptation in brown trout (*Salmo trutta* L.): Footprints of selection at microsatellite DNA loci. Heredity, 106(3), 488–499. 10.1038/hdy.2010.164 21224872 PMC3131969

[jfb70247-bib-0039] Metcalfe, N. B. , Taylor, A. C. , & Thorpe, J. E. (1995). Metabolic rate, social status and life‐history strategies in Atlantic salmon. Animal Behaviour, 49(2), 431–436. 10.1006/anbe.1995.0056

[jfb70247-bib-0040] Nevoux, M. , Finstad, B. , Davidsen, J. G. , Finlay, R. , Josset, Q. , Poole, R. , Höjesjö, J. , Aarestrup, K. , Persson, L. , Tolvanen, O. , & Jonsson, B. (2019). Environmental influences on life history strategies in partially anadromous brown trout (*Salmo trutta*, Salmonidae). Fish and Fisheries, 20(6), 1051–1082. 10.1111/faf.12396

[jfb70247-bib-0041] Östergren, J. , Lundqvist, H. , & Nilsson, J. (2011). High variability in spawning migration of sea trout, *Salmo trutta*, in two northern Swedish rivers. Fisheries Management and Ecology, 18(1), 72–82. 10.1111/j.1365-2400.2010.00774.x

[jfb70247-bib-0042] Páez, D. J. , Morrissey, M. , Bernatchez, L. , & Dodson, J. J. (2010). The genetic basis of early‐life morphological traits and their relation to alternative male reproductive tactics in Atlantic salmon. Journal of Evolutionary Biology, 23(4), 757–768. 10.1111/j.1420-9101.2010.01941.x 20149020

[jfb70247-bib-0043] Palm, D. , Losee, J. , Andersson, S. , Gustav, H. , Holmgren, A. , & Spong, G. (2023). Dispersal of brown trout (*Salmo trutta* L.) fry in a low gradient stream—Implications for egg stocking practices. River Research and Applications, 39(4), 790–796. 10.1002/rra.4093

[jfb70247-bib-0044] Phillips, B. L. , & Perkins, T. A. (2019). Spatial sorting as the spatial analogue of natural selection. Theoretical Ecology, 12(2), 155–163. 10.1007/s12080-019-0412-9

[jfb70247-bib-0045] Podgorniak, T. , Blanchet, S. , De Oliveira, E. , Daverat, F. , & Pierron, F. (2016). To boldly climb: Behavioural and cognitive differences in migrating European glass eels. Royal Society Open Science, 3(1), 150665. 10.1098/rsos.150665 26909192 PMC4736947

[jfb70247-bib-0046] Reid, J. M. , Souter, M. , Fenn, S. R. , Acker, P. , Payo‐Payo, A. , Burthe, S. J. , Wanless, S. , & Daunt, F. (2020). Among‐individual and within‐individual variation in seasonal migration covaries with subsequent reproductive success in a partially migratory bird. Proceedings of the Royal Society B: Biological Sciences, 287, 20200928. 10.1098/rspb.2020.0928 PMC7423652 32693718

[jfb70247-bib-0047] Rougemont, Q. , Xuereb, A. , Dallaire, X. , Moore, J. , Normandeau, E. , Perreault‐Payette, A. , Bougas, B. , Rondeau, E. B. , Withler, R. E. , Van Doornik, D. M. , Crane, P. A. , Naish, K. A. , Garza, J. C. , Beacham, T. D. , Koop, B. F. , & Bernatchez, L. (2023). Long‐distance migration is a major factor driving local adaptation at continental scale in Coho salmon. Molecular Ecology, 32(3), 542–559. 10.1111/mec.16339 35000273

[jfb70247-bib-0048] Sahashi, G. , & Morita, K. (2013). Migration costs drive convergence of threshold traits for migratory tactics. Proceedings of the Royal Society B: Biological Sciences, 280(1773), 20132539. 10.1098/rspb.2013.2539 PMC3826239 24197418

[jfb70247-bib-0049] Serbezov, D. , Bernatchez, L. , Olsen, E. M. , & Vøllestad, L. A. (2010). Quantitative genetic parameters for wild stream‐living brown trout: Heritability and parental effects. Journal of Evolutionary Biology, 23(8), 1631–1641. 10.1111/j.1420-9101.2010.02028.x 20524953

[jfb70247-bib-0050] Stamps, J. A. (2007). Growth‐mortality tradeoffs and ‘personality traits’ in animals. Ecology Letters, 10(5), 355–363. 10.1111/j.1461-0248.2007.01034.x 17498134

[jfb70247-bib-0051] Stelkens, R. B. , Jaffuel, G. , Escher, M. , & Wedekind, C. (2012). Genetic and phenotypic population divergence on a microgeographic scale in brown trout. Molecular Ecology, 21(12), 2896–2915. 10.1111/j.1365-294X.2012.05581.x 22554245

[jfb70247-bib-0052] Strøm, J. F. , Jensen, J. L. A. , Nikolopoulos, A. , Nordli, E. , Bjørn, P. A. , & Bøhn, T. (2021). Sea trout *Salmo trutta* in the subarctic: Home‐bound but large variation in migratory behaviour between and within populations. Journal of Fish Biology, 99(4), 1280–1291. 10.1111/jfb.14832 34184272

[jfb70247-bib-0053] Taylor, E. B. , & McPhail, J. D. (1985). Variation in body morphology among British Columbia populations of Coho Salmon, *Oncorhynchus kisutch* . Canadian Journal of Fisheries and Aquatic Sciences, 42(12), 2020–2028. 10.1139/f85-249

[jfb70247-bib-0054] Vøllestad, L. A. , Serbezov, D. , Bass, A. , Bernatchez, L. , Olsen, E. M. , & Taugbøl, A. (2012). Small‐scale dispersal and population structure in stream‐living brown trout (*Salmo trutta*) inferred by mark–recapture, pedigree reconstruction, and population genetics. Canadian Journal of Fisheries and Aquatic Sciences, 69(9), 1513–1524. 10.1139/f2012-073

[jfb70247-bib-0055] Yamahira, K. , & Conover, D. O. (2002). Intra‐ vs. interspecific latitudinal variation in growth: Adaptation to temperature or seasonality? Ecology, 83(5), 1252–1262. 10.1890/0012-9658(2002)083[1252:IVILVI]2.0.CO;2

[jfb70247-bib-0056] Yamahira, K. , Kawajiri, M. , Takeshi, K. , & Irie, T. (2007). Inter‐ and intrapopulation variation in thermal reaction norms for growth rate: Evolution of latitudinal compensation in ectotherms with a genetic constraint. Evolution, 61(7), 1577–1589. 10.1111/j.1558-5646.2007.00130.x 17598741

[jfb70247-bib-0057] Zimmer, M. P. , & Power, M. (2006). Brown trout spawning habitat selection preferences and redd characteristics in the Credit River, Ontario. Journal of Fish Biology, 68(5), 1333–1346. 10.1111/j.0022-1112.2006.00995.x

